# Neuronal networks underlying ictal and subclinical discharges in childhood absence epilepsy

**DOI:** 10.1007/s00415-022-11462-8

**Published:** 2022-11-12

**Authors:** Ami Kumar, Ekaterina Lyzhko, Laith Hamid, Anand Srivastav, Ulrich Stephani, Natia Japaridze

**Affiliations:** 1 Department of Neuropediatrics, Children’s Hospital, University Medical Center Schleswig-Holstein, University of Kiel, Kiel, Germany; 2 Faculty of Mathematics and Natural Sciences, University of Kiel, Kiel, Germany; 3 Department of Neurology, Columbia University Irving Medical Center, New York, USA; 4 Institute of Medical Psychology and Medical Sociology, University of Kiel, Kiel, Germany; 5 Department of Psychiatry and Psychotherapy, Jena University Hospital, Jena, Germany

**Keywords:** Seizures, Childhood absence epilepsy, Ictal discharges, Subclinical discharges, EEG, Source localization, eLORETA, Functional connectivity, Imaginary part of coherency

## Abstract

Childhood absence epilepsy (CAE), involves 3 Hz generalized spikes and waves discharges (GSWDs) on the electroencephalogram (EEG), associated with ictal discharges (seizures) with clinical symptoms and impairment of consciousness and subclinical discharges without any objective clinical symptoms or impairment of consciousness. This study aims to comparatively characterize neuronal networks underlying absence seizures and subclinical discharges, using source localization and functional connectivity (FC), to better understand the pathophysiological mechanism of these discharges. Routine EEG data from 12 CAE patients, consisting of 45 ictal and 42 subclinical discharges were selected. Source localization was performed using the exact low-resolution electromagnetic tomography (eLORETA) algorithm, followed by FC based on the imaginary part of coherency. FC based on the thalamus as the seed of interest showed significant differences between ictal and subclinical GSWDs (*p* < 0.05). For delta (1–3 Hz) and alpha bands (8–12 Hz), the thalamus displayed stronger connectivity towards other brain regions for ictal GSWDs as compared to subclinical GSWDs. For delta band, the thalamus was strongly connected to the posterior cingulate cortex (PCC), precuneus, angular gyrus, supramarginal gyrus, parietal superior, and occipital mid-region for ictal GSWDs. The strong connections of the thalamus with other brain regions that are important for consciousness, and with components of the default mode network (DMN) suggest the severe impairment of consciousness in ictal GSWDs. However, for subclinical discharges, weaker connectivity between the thalamus and these brain regions may suggest the prevention of impairment of consciousness. This may benefit future therapeutic targets and improve the management of CAE patients.

**Supplementary Information:**

The online version contains supplementary material available at 10.1007/s00415-022-11462-8.

## Introduction

Childhood absence epilepsy (CAE) is a generalized, genetic form of epilepsy, characterized by absence seizures and electroencephalographic (EEG) recordings with generalized spikes and waves discharges (GSWDs). The seizures are often referred to as ictal GSWDs, and are associated with severe impairment of consciousness [1–3]. The interictal EEG of CAE patients also show periodic bursts of subclinical GSWDs, identical to ictal patterns, but without any impairment of consciousness or behavioral alterations. Interestingly, even though there are crucial clinical differences between absences associated EEG discharges and subclinical EEG discharges, it is impossible to differentiate them electrographically and ultimately only a direct clinical testing during EEG recordings can reliably distinguish seizures. It is not clear as to why some GSWDs are accompanied by transient impairment of consciousness and others not.

In our previous study, we addressed this topic and focused on exploring differences on the sensor level between ictal and subclinical GSWDs using routine EEG [4]. We have demonstrated that ictal discharges have higher spectral power and longer durations than subclinical GSWDs. Guo et al. had shown similar results using the simultaneous acquisition of EEG and functional magnetic resonance imaging (fMRI). It can be speculated that these differences could reflect a more intensive and larger neuronal activity, which may cause a transient impairment of consciousness. Guo et al. concluded, however, that behavioural impairment from longer seizures is not simply due to prolonged brain dysfunction, but rather that some seizures have more severe physiological abnormalities already at, or before seizure onset [5]. This idea is supported by studies that have demonstrated that fMRI changes can precede seizures by several seconds [6].

Numerous experimental studies have shown that highly synchronized cortico-subcortical networks are involved in the generation of GSWDs [7–11]. Functional neuroimaging studies in patients with idiopathic generalized epilepsies demonstrated that the thalamus, caudate nuclei, and default mode network (DMN) areas are involved in GSWD paroxysms [12–17]. In addition, EEG-fMRI studies regarding absence seizures show activation of the thalamus and deactivation of regions such as medial frontal, medial parietal, anterior, and posterior cingulate, including certain components of the DMN [13, 17]. It is noteworthy that certain components of the DMN overlap with the consciousness system [18, 19].

Since techniques such as fMRI have a low temporal resolution, it becomes difficult to use this to demonstrate the temporal follow up between structures involved within absence seizures. EEG on the other hand has the advantage of millisecond temporal resolution and is better suited for analysing these networks. However, the static bioelectromagnetic inverse problem poses a challenge of low spatial resolution, which becomes especially important for deep brain source imaging [20]. Nonetheless, recent developments regarding EEG inverse and forward solutions have significantly improved the precision of source localization, especially in combination with functional connectivity, and numerous studies have been able to localize deep brain sources [21–24].

The aim of this study was to perform a comparative investigation of neuronal networks and their dynamics during ictal and subclinical GSWDs. We hypothesize that the direct comparison of these two types of events can help distinguish the critical nodes or dynamics of brain networks responsible for the cognitive impairment seen during absence seizures and help us to better understand the intricate mechanisms of the consciousness system. Further on, this study aimed to provide an insight into the methodology of EEG based source-level connectivity.

## Materials and methods

### Subjects

All CAE patients were recruited retrospectively from the North German Epilepsy Centre for Children and Adolescents, Schwentinental-Raisdorf, during 2008–2018. Initially, the EEGs from 26 patients were analyzed. For all patients, the CAE diagnosis was based on the clinical and electrographic criteria proposed by Panayiotopoulos CP., in 2005 [25]. Only those patients were selected for further analysis, who fulfilled the study criteria. The study criteria involved firstly, patients being tested for impairment of consciousness, consistently and continuously during the GSWDs, and secondly, the occurrence of both ictal and subclinical GSWDs in the EEG recordings of these patients. Based on this, 12 patients (7 males, 5 females, mean age of 7.3 ± 1.5) who fulfilled the inclusion criteria were recruited for this study. The selection criteria as well as the characteristics of this cohort are described in detail in our previous publication [4]. The study received approval from the ethics committee at the faculty of medicine, University of Kiel (No. D 456/19).

### EEG recordings

The routine EEGs were recorded based on the standard international 10–20 system, with 31 scalp electrodes. The recording system used was from Neurofile (Natus Europe GmbH, Germany). All recordings consisted of patients being awake. The sampling rate was 512 Hz with the impedance being below 10 kOhms, and the reference electrode was located between Fz and Cz.

### Data analysis

#### Selection of ictal and subclinical GSWDs

All events were differentiated by experienced epileptologists via video EEG recordings. Events were classified into absences and subclinical GSWDs based on electrographic features and clinical signs of presence of consciousness during GSWDs. EEG segments in which the presence of consciousness was not reliably tested were excluded from the study. A detailed description of the selection criteria of absences and subclinical discharges has been given in our previous publication [4]. A total of 45 ictal discharges and 42 subclinical discharges were selected for analysis. For both ictal and subclinical GSWDs, three time segments were analyzed. The full duration of the ictal/subclinical discharge was selected alongside, 3 s pre-ictal/subclinical and 3 s post-ictal/subclinical.

#### Pre-processing

Pre-processing was performed using the FieldTrip toolbox [26]. The EEG data were bandpass filtered within the range of 0.1–31 Hz. Artifacts were removed using independent component analysis (ICA) and a common average reference was used to re-reference the EEG channels. The data were normalized using a Z-score normalization, and the selected segments were further divided into 1 s segments. For these segments, the mean and trend were removed using a general linear model. All further analyses were performed for a frequency range of 1–30 Hz including the bands delta (1–3 Hz), theta (4–7 Hz), alpha (8–12 Hz), and beta (13–30 Hz). The publication of [4] describes this methodology in more detail.

#### Source localization

Source localization was performed using the MEG and EEG Toolbox of Hamburg (METH, https://www.nitrc.org/projects/meth/) and the FieldTrip software. To reconstruct the current density distribution that describes the onset zone of a specific activity observed on the scalp, the EEG forward and inverse problems need to be solved. The forward problem [27] is described as the computation of scalp potentials for a known source and a known head model. The resulting lead field matrix (LFM), which describes the mapping between the source space in the brain and the EEG electrodes, alongside specified brain grids, meshes, or discretization is important for the EEG inverse solution.

In this study, the realistic volume conductor or head model was used in the numerical solution of the forward problem via the boundary element method (BEM). The volume conductor model contains information regarding the geometry and the conductivity of the head tissues. The description of the geometry was created using a standard T1-weighted MRI, which is available in Fieldtrip, followed by the segmentation of the head MRI into three compartments, each corresponding to the scalp, skull and brain. From the segmented MRI, a standard head model is created. For further calculations, the toolbox METH was used. The conductivity values of the head tissues are taken from the literature. Using information regarding the electrode positions and the volume conductor, certain structural calculations were done, and this information was combined into a particular data structure. The positions of the electrodes are according to the Montreal Neurological Institute (MNI) coordinates. Further, the LFM was calculated, and a brain grid was selected for further analysis. The grid comprises of voxels (5003), where each voxel represents a dipole in the brain with three orthogonal directions.

Following this, a solution of the EEG inverse problem was selected. The solution of the EEG inverse problem involves the estimation of the unknown neural current sources from the measured scalp potentials [21]. This neural activity can be modelled as a distribution of current dipoles. By using a linear transformed spatial filter, the power or coherence at any given location of the brain can be computed [28]. The EEG inverse method called the eLORETA algorithm [29] was used to construct a spatial filter for each voxel, and also for all orthogonal dipole directions such that for a given voxel the filter is an N × 3 matrix for N channels. This helps in identifying the maximum spatial power or coherence for a specific frequency band. The 3-dimensional filters for eLORETA are calculated using the LFM, with output being tensors of size N × M × 3, for N channels (31), M voxels (5003), and 3 orthogonal dipole directions. As a second step, the sensor-level cross spectrum was calculated for 1-s-long segments and for a frequency range of 1–30 Hz with 1 Hz step. The input data consists of a T × N matrix for T time points and N channels. The resulting cross-spectrum was averaged over segments and frequency ranges delta (1–3 Hz), theta (4–7 Hz), alpha (8–12 Hz), and beta (13–30 Hz). The output is a sensor-level cross-spectrum matrix of the size N × N × P, for N channels and P frequencies for each segment.

Furthermore, the source direction was estimated using the 3-dimensional filter and the sensor-level cross-spectral matrix for each voxel, based on the direction that maximizes the power for each voxel. The output consists of the power at each voxel, the dipole direction found for each voxel, and a 1-dimensional filter for each voxel.

#### Functional connectivity based on the imaginary part of coherency

The toolbox METH was used for calculating the source-level imaginary part of coherency. Based on the seed of interest in the brain, the resulting connectivity patterns for ictal and subclinical discharges were mapped.

The 1-dimensional filter obtained during source localization was used to calculate the cross-spectrum at the source level, by applying the 1-dimensional filter on the sensor-level cross-spectral matrix. The following calculation was used: *A1*^*T*^* x cross-spectrum x A1.*

Where A1 is the 1-dimensional filter, and the cross-spectral matrix is from the sensor space.

Following this, the coherence was calculated from the source-level cross-spectrum, with the output being a coherency matrix consisting of the real and imaginary parts of coherency. This matrix was further used to obtain the coherency values corresponding to the thalamus as the seed and to extract the absolute value of the imaginary part of coherency.

Since the results of the connectivity analysis may be influenced by factors such as volume conduction, usage of standard MRI, and low-density EEG, we further highlight the most significant brain regions using the automated anatomical labeling (AAL) atlas [30] to parcellate the brain regions and estimate the percentage of the volume of each brain region that is involved in the statistically significant differences in connectivity. A threshold value was used to exclude the brain regions, in which less than 30% of the total volume of structures were included. We then inspected the brain regions and tried to discover meaningful networks and influences on brain function. Further details are described in Appendix 2 of the Supplementary information.

### Statistical analysis

The statistical significance was identified using a 2-way within-subject cluster-based permutation ANOVA (Analysis of variance) [31, 32], which is based on non-parametric statistics and is available in the FieldTrip toolbox. The significance probability was estimated based on a Monte Carlo permutation test with a cluster-based approach [33]. The permutation test avoids assumptions about the data being normally distributed, and solves the multiple comparisons problem by cluster correction. The Monte Carlo test consisted of 2000 random permutations and a *p* value (*p* ≤ 0.05) was calculated. The two within-subject factors consisted of factor *‘group’* (ictal, subclinical) and factor *‘time’* (pre-ictal/subclinical, during-ictal/subclinical, and post-ictal/subclinical). For significant ANOVA results (*p* ≤ 0.05) having more than one pair of comparisons, a post-hoc *t*-test was performed. This analysis was performed for all frequency bands within the range of 1–30 Hz.

### Visualization

Fieldtrip was used for visualizing the source localization and connectivity analysis results. Firstly, using certain fieldtrip functions the results of the source activity and statistical maps were interpolated onto the MRI voxels. A mask from statistics is used based on the significant *p* value generated (*p* < 0.05) by the statistical analysis. We used the source activity together with the anatomical MRI and the mask obtained from statistics. For connectivity results, this procedure was used as well. The results were visualized as 2D axial slices of the brain along with the plotted functional data, and as images containing axial, sagittal, and coronal slices. For anatomical labelling, the AAL atlas was used.

## Results

### Subjects

From the routine EEGs of 12 patients, 45 ictal GSWDs and 42 subclinical GSWDs were selected. The mean duration of ictal discharges was 8.4 s (SD 3.4), while for subclinical discharges it was 4.1 s (SD 2.4). All patients were on standard CAE medication at the time of EEG recordings. The clinical and demographic data of the patients is described in our previous publication [4].

### Source localization

**Fig. 1:**
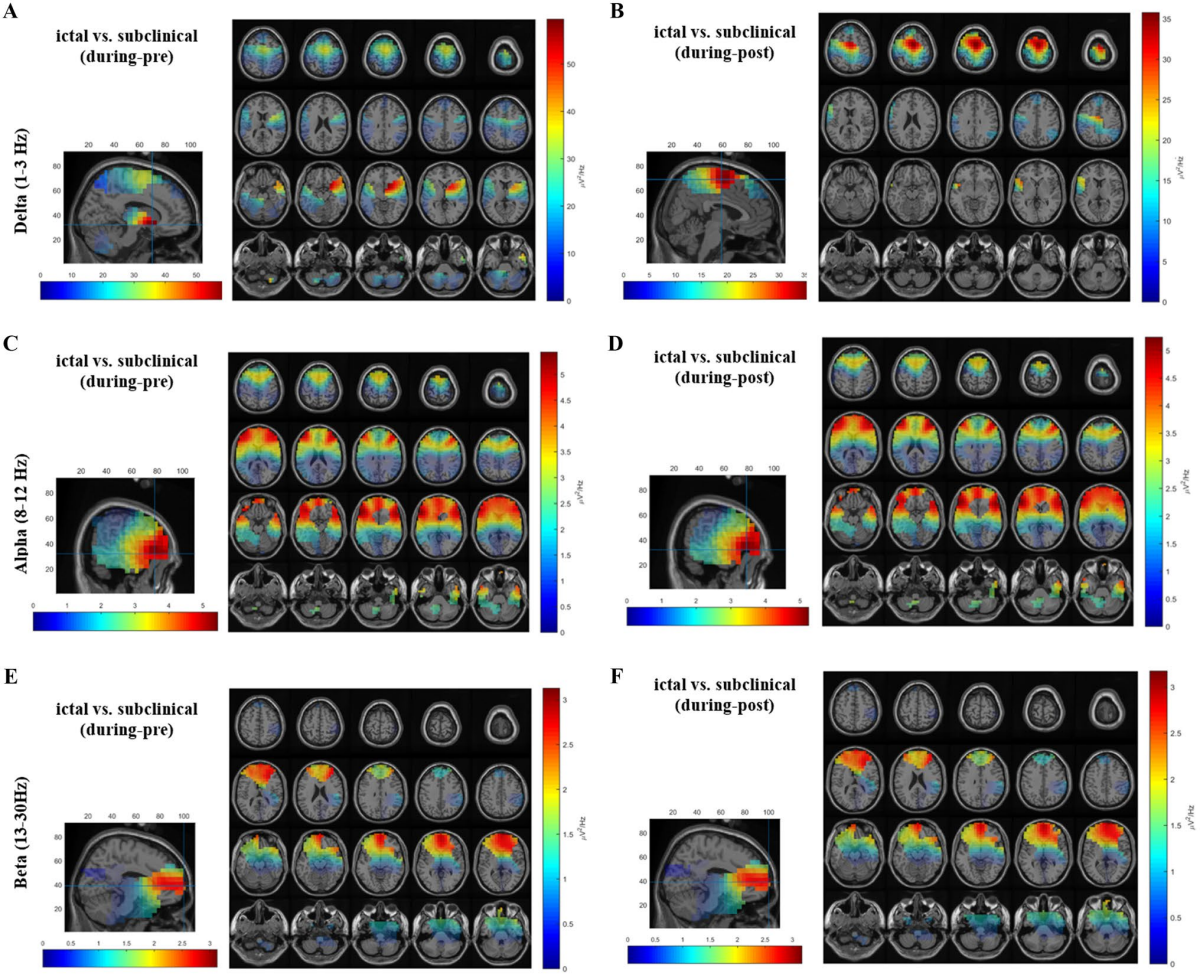
Source localization results for interaction effect. Significant results for interaction effect are depicted for all frequency bands (delta, alpha and beta) using EEG data of ictal and subclinical discharges during the transition periods (pre-during and during-post). For all frequency bands ictal GSWDs have stronger source power as compared to subclinical GSWDs. Figures demonstrate the significant source power, as seen on sagittal and axial brain slices. **A** Delta band, pre-during transition interval depicting the source-level maximum power in deep subcortical caudate and putamen regions, along with the thalamus. **B** Delta band, during-post transition interval, where the source-level maximum power is localized in the supplementary motor region. **C** Alpha band, pre-during transition period, with the source-level maximum power in the frontal inferior orbital left hemisphere. **D** Alpha band, during-post transition interval also with the source-level maximum power localized in the frontal inferior orbital left hemisphere. **E** Beta band, pre-during transition interval, with source-level maximum power localized in the frontal superior right hemisphere. **F** Beta band during-post transition period, also with the source-level maximum power localized in the frontal superior right hemisphere

For the time effect, significant differences were revealed for all frequency bands, with the during-ictal/subclinical interval having stronger source power compared to the pre- and post-ictal/subclinical intervals (Supplementary Fig. 1). As for the main effect of group, all frequency bands except the theta band demonstrated significant differences, with ictal GSWDs having higher source power as compared to subclinical GSWDs (Supplementary Fig. 2). Furthermore, compared to subclinical GSWDs, ictal GSWDs showed a significant interaction effect for delta (Fig. 1A, B), alpha (Fig. 1C, D) and beta (Fig. 1E, F) frequency bands that revealed stronger source power for transition intervals, pre-during, and during-post. A detailed description of source analysis results is given in (Supplementary information Appendix 1). The maximums of the source differences, for all frequency bands and conditions, have been listed in Table 1.

**Table 1:** Source analysis results

Conditions	Source maximum							
	Delta	*p* values	Theta	*p* values	Alpha	*p* values	Beta	*p* values
Effect of time								
During vs. pre	Rectus L, R	0.001	Rectus R, L	0.001	Rectus L, R	0.001	Frontal inferior orbital R	0.001
During vs. post	Rectus L, R Caudate L	0.001	Rectus R, L	0.001	Rectus L, R	0.001	Frontal inferior orbital R	0.001
Effect of group								
Ictal vs. subclinical	Rectus L	0.002	**–**	**–**	Frontal inferior orbital L	0.01	Frontal superior medial R	0.04
Interaction effect								
Ictal vs. subclinical (during-pre)	Caudate R Putamen R	0.01	**–**	**–**	Frontal inferior orbital L	0.005	Frontal superior medial R	0.01
Ictal vs. subclinical (during-post)	Supplementary motor area R	0.04	–	–	Frontal inferior orbital L	0.008	Frontal superior medial R	0.01

*R* right, *L* left

As described, the maximum differences were localized in the subcortical regions of caudate and putamen, alongside lower differences of power seen in the thalamus for the delta band, pre-during transition period (Fig. 1A).

### Functional connectivity

Based on the literature regarding generalized seizures, there are abundant descriptions of the thalamus being one of the most important brain regions involved in seizure onset and seizure propagation [17, 34, 35]. Therefore, in this study, the thalamus was used as the seed of interest for subsequent source-level connectivity analyses for all considered frequency bands (1–30 Hz). Our data were also checked for consistency with earlier studies regarding increased thalamic activity during ictal discharges in the delta range. The results of statistical comparisons via a *t*-test showed higher power in the subcortical regions (right caudate, right pallidum, right putamen, and right thalamus) for delta band (1–3 Hz) during ictal compared to subclinical discharges (Supplementary Fig. 3). Although the maximum of the significant differences (right caudate) is, unlike our expectations, not located at the thalamus, we suppose that this is a shift in the source location caused by a localization bias. This bias may be caused by using a standard MRI, the volume conduction effect, or the use of low-density EEG.

The connectivity analysis results based on the thalamus as the seed of interest were investigated for all frequency bands (1–30 Hz):

#### Delta band

**Fig. 2:**
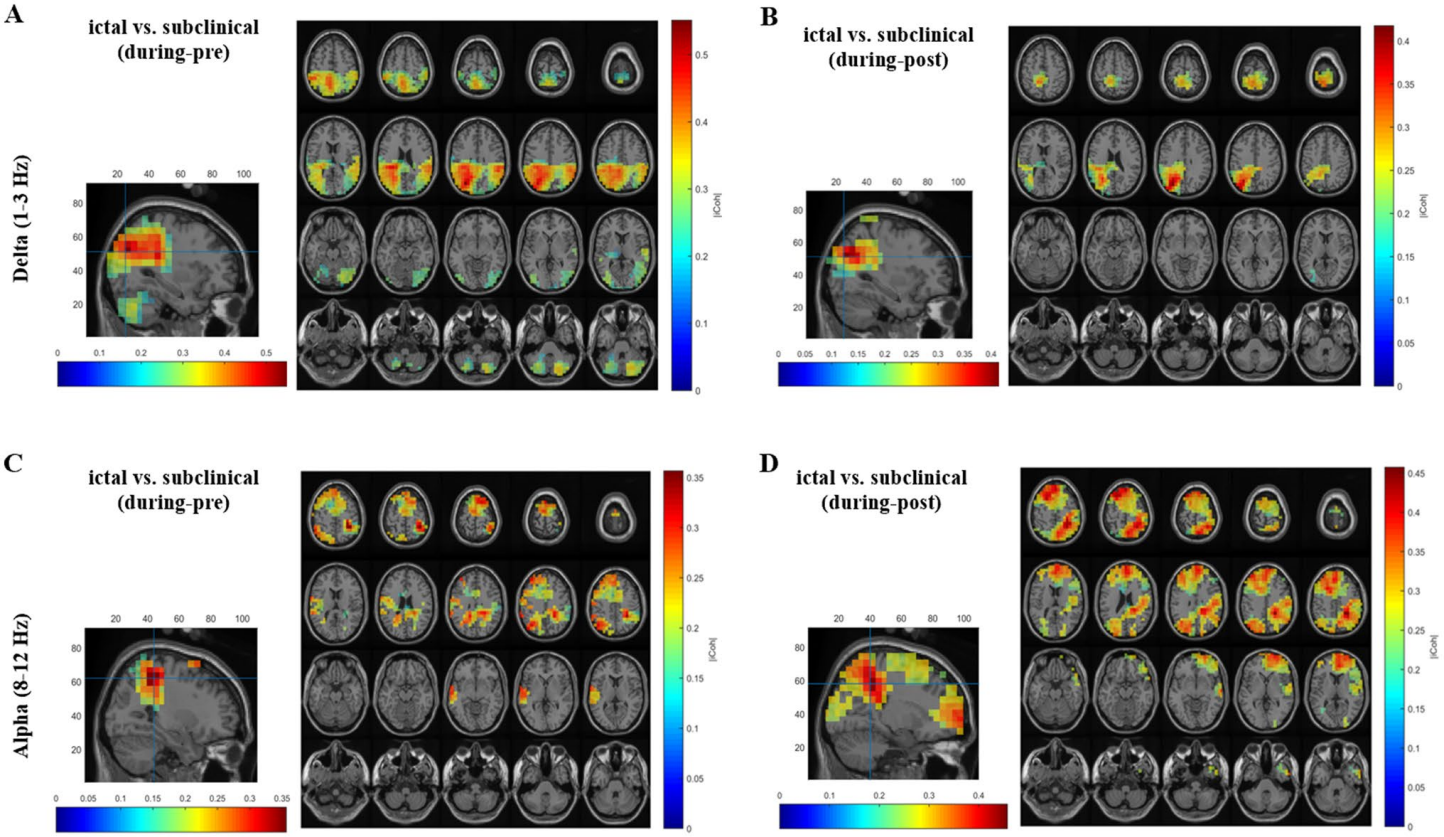
Functional connectivity results for interaction effect. Significant results for the interaction effect were seen for the delta and alpha frequency bands. With the thalamus as the seed of interest, the transition intervals for ictal discharges pre-during and during-post, had significantly stronger imaginary part of coherency to other brain regions as compared to subclinical discharges. The significant brain regions have been listed in Table 2. The significant coherence maps can be seen on sagittal and axial brain slices. **A** Delta band, pre-during transition interval **B** Delta band, during-post transition interval **C** Alpha band, pre-during transition interval **D** Alpha band, during-post transition interval

2-way ANOVA demonstrated a significant time and group effect, as well as a significant interaction effect. Post hoc tests for the main effect of time revealed that during-ictal/subclinical time interval has a stronger imaginary part of coherency compared to the pre- and post-ictal/subclinical time intervals (Supplementary Fig. 4). The maximum coherence difference for the time effect was seen in the cingulum posterior right, precuneus right, for pre- to during-ictal/subclinical interval (*p* < 0.001) and for during- to post-ictal/subclinical interval (*p* < 0.001). For the main effect of group, ictal discharges had a stronger imaginary part of coherency compared to subclinical discharges, with the maximum coherence difference seen in the supramarginal right, and angular right regions for ictal vs. subclinical discharges (*p* = 0.02) (Supplementary Fig. 5). The post hoc test for the interaction effect revealed that the ictal transition periods pre-during and during-post have a stronger imaginary part of coherency compared to equivalent transition periods of subclinical discharges (Fig. 2A, B). These significant coherence differences were seen in brain regions described in Table 2.

**Table 2:** Functional connectivity using the thalamus as a seed

Frequency bands	Ictal vs. Subclinical Interaction effect (group × time)	*p* values	FC using the thalamus as a seed
Delta band (1–3 Hz)	During-pre	0.006	Cingulum posterior L, R Precuneus L Angular L, R Supramarginal L, R Occipital mid L, R
	During-post	0.04	Cingulum posterior L Supramarginal L Precuneus L Cuneus L Angular L Occipital mid L
Theta band (4–7 Hz)	-No significance-		
Alpha band (8–12 Hz)	During-pre	0.01	Postcentral R Parietal Inferior R Supplementary motor area R Frontal superior R Cingulum mid L Frontal superior medial L Frontal mid L Parietal inferior L, R Angular L Precuneus L
	During-post	0.003	Parietal Inferior L, R Angular L, R Parietal Superior R Precuneus R Cuneus R Supramarginal R Occipital superior R Frontal mid L Frontal superior L Supplementary motor area L Frontal superior medial L
Beta band (13–30 Hz)	-No significance-		

*R* right, *L* left

#### Theta band

For theta band, only a significant time effect was seen, while no significant group or interaction effects were seen. Post hoc tests for theta band demonstrated for the main effect of time that, the during-ictal/subclinical interval has a stronger imaginary part of coherency compared to pre- and post- ictal/subclinical time intervals. These differences were widespread in the frontal-central, parietal right, and left, occipital right, and left regions and the maximum coherence difference could be observed in the precuneus right region for the interval of pre- to during- ictal/subclinical (*p* < 0.001) and in the cingulum middle, cingulum posterior, and precuneus left regions for during- to post-ictal/subclinical interval (*p* < 0.001) (Supplementary Fig. 4).

#### Alpha band

For alpha band 2-way ANOVA showed a significant time effect but no significant group effect. Further, there was a significant interaction effect. The post hoc test for the main effect of time revealed that the during-ictal/subclinical time interval has a stronger imaginary part of coherency compared to pre- and post-ictal/subclinical time intervals. The coherence was seen to be widespread in the brain regions except for the mid-central region. The maximum coherence difference could be observed in angular right region for the time interval pre- to during-ictal/subclinical (*p* < 0.001) and for during- to post-ictal/subclinical interval (*p* < 0.001) it could be observed in the parietal inferior, the angular, and supramarginal right regions (Supplementary Fig. 4). The post hoc test for the interaction effect revealed that the coherence for ictal transition periods from pre-during and from during-post intervals was stronger compared to the transition periods of subclinical discharges (Fig. 2C, D). Brain regions showing significant coherent differences are described in Table 2.

#### Beta band

2-way ANOVA for beta band revealed a significant time effect but no significant group or interaction effects. The post hoc test for the main effect of time demonstrated similar results to other frequency bands. The during-ictal/subclinical time interval had a stronger imaginary part of coherency compared to pre- and post-ictal/subclinical time intervals. The maximum coherence difference for the time interval pre- to during-ictal/subclinical (*p* < 0.001) was observed in the angular right region, and for the during- to post-ictal/subclinical time interval (*p* < 0.001) it was observed in the parietal inferior, angular, and supramarginal right regions (Supplementary Fig. 4).

**Fig. 3:**
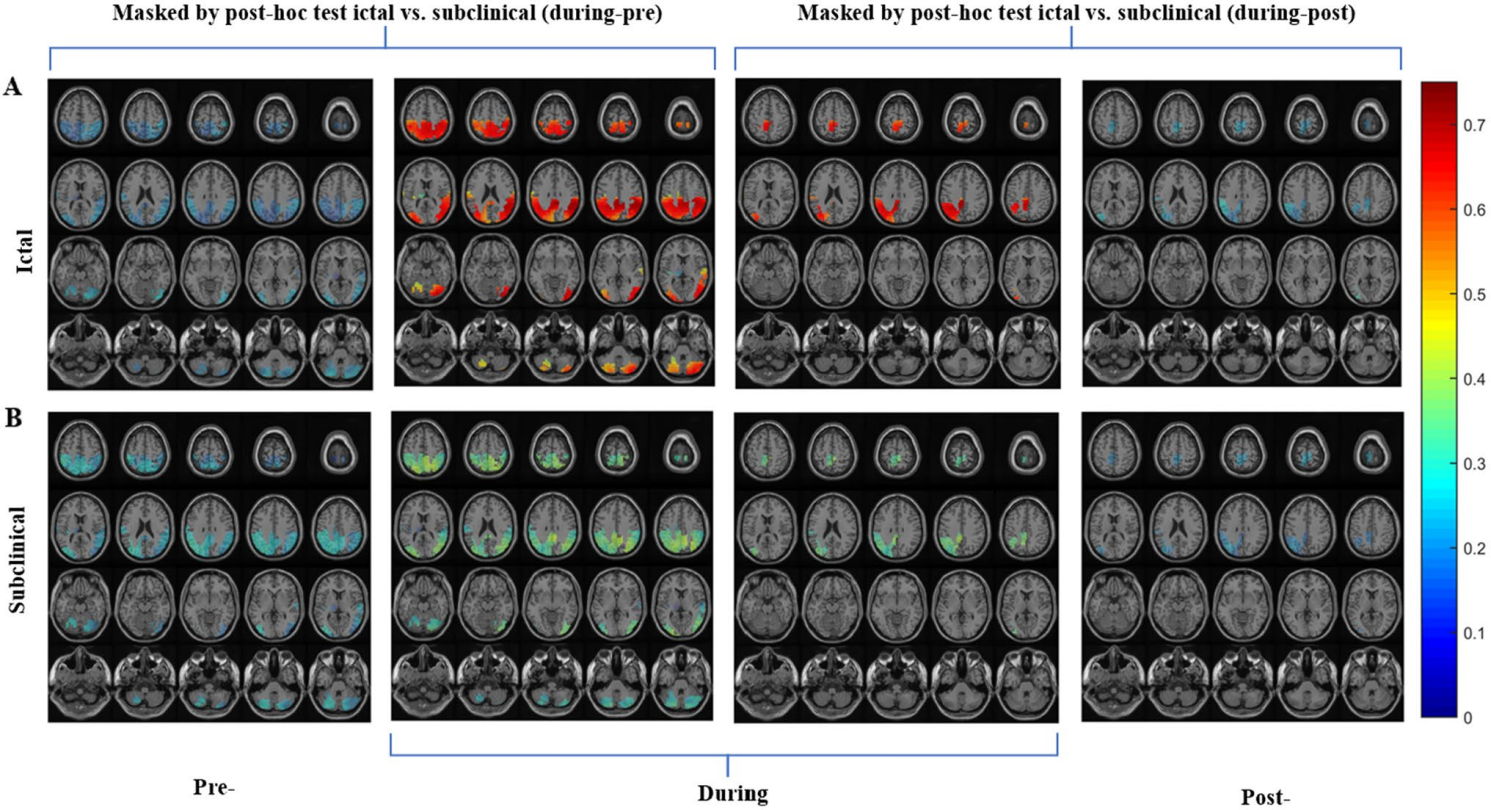
Delta Band functional connectivity results with 30% thresholding for interaction effect. Imaginary part of coherency for delta band (1–3 Hz) averaged over patients for each group (ictal and subclinical) and time interval (pre-, during and post-), using two statistical masks derived from ANOVA. **A** Dynamical network changes for ictal discharges for all time intervals, depicting the connectivity being strong during-ictal discharges in the posterior brain regions, and the pre- and post- intervals having weaker connectivity. **B** Network changes as seen for subclinical discharges, consisting of weaker connectivity for all time intervals

**Fig. 4:**
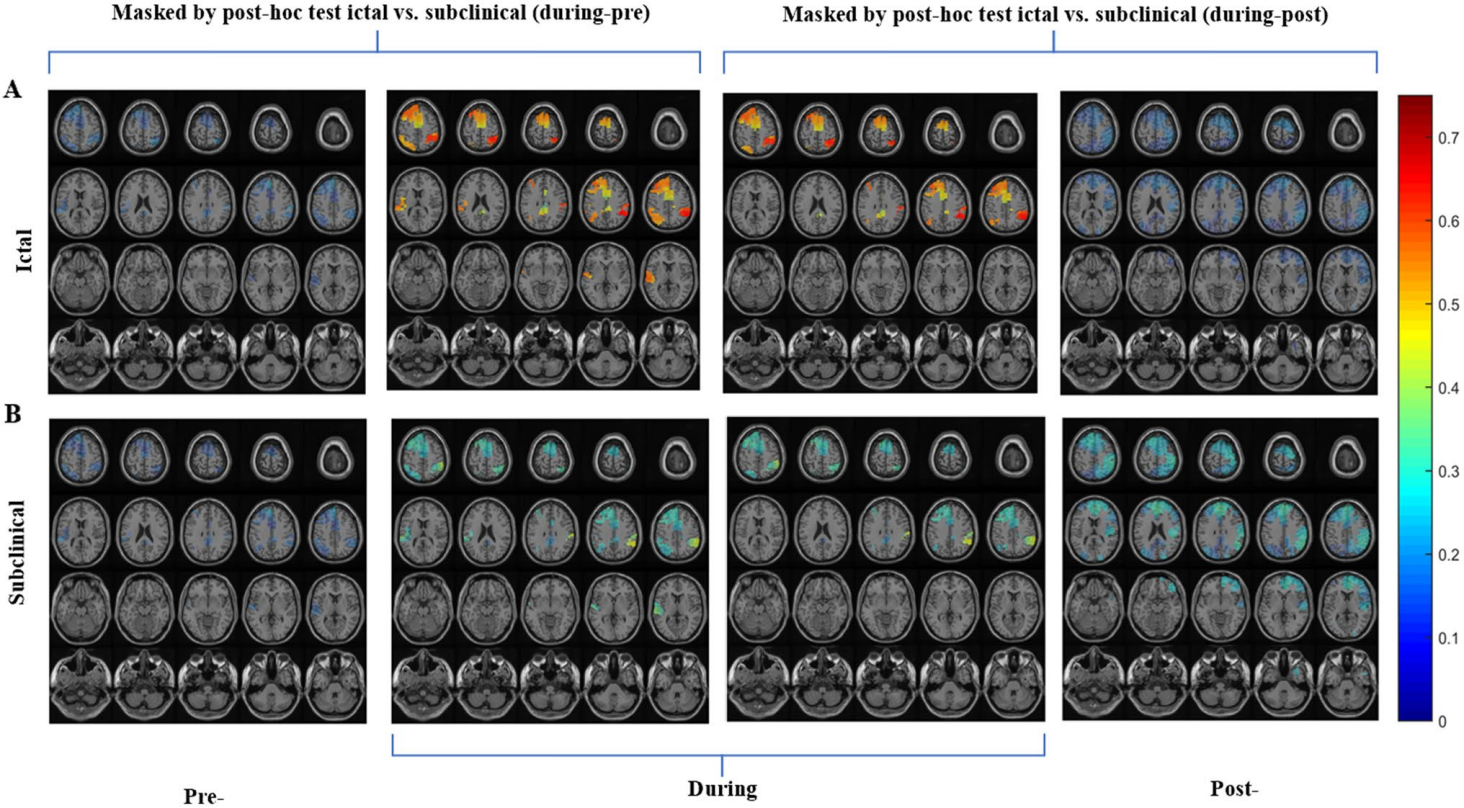
Alpha band functional connectivity results with 30% thresholding for interaction effect. Imaginary part of coherency for alpha band (8–12 Hz) averaged over patients for each group (ictal and subclinical) and time interval (pre-, during and post-), using two statistical masks derived from ANOVA. **A** Network distribution for ictal discharges demonstrating stronger connectivity during-ictal discharges in frontal and posterior brain regions, and the pre- and post-intervals having weaker connectivity. **B** Network distribution for subclinical discharges depicting weaker connectivity for all time intervals

Furthermore, for the frequency bands showing a significant interaction effect (delta and alpha bands), the FC was also evaluated by thresholding the brain regions to 30%, to better observe the underlying dynamical network. For this, the imaginary part of coherency was averaged over patients for each group (ictal and subclinical) and time interval (pre-, during- and post-). Two statistical masks, estimated by ANOVA for the interaction effect were applied. For both masks, a threshold value was used to exclude brain regions, in which there is less than 30% statistically significant volume. The first mask was estimated by the comparison of ictal vs. subclinical for the transition periods pre- to during, and the second mask was estimated by the comparison of ictal vs. subclinical for the transition period during- to post. Such visualization also avoids the erroneous interpretation of the results for the transition periods because of the double use of subtraction. By doing so, the dynamical changes of networks in delta and alpha frequency bands can be clearly depicted by the values of the imaginary part of coherency for ictal and subclinical discharges for three time intervals (pre-, during- and post). For delta band (Fig. 3) the network structure is similar for both masks, however during-ictal discharges the network is very strongly activated as compared to subclinical discharges. Additionally, the network during subclinical discharges has similar low activations for pre- and post-intervals of ictal and subclinical discharges. A detailed list of the significant brain regions involved in the brain network for the transition periods has been listed in Supplementary Tables 1 and 2. Further, similar dynamics were observed for alpha band (Fig. 4), with a strong network activation during ictal discharges compared to the activation during subclinical discharges. Similar low activation values were observed for both types of discharges in pre- and post-intervals. The relevant brain regions in the network dynamics have been listed in Supplementary Tables 3 and 4. Further details have been described in Appendix 2 of the Supplementary information.

## Discussion

In this study, we focused on exploring neuronal networks underlying GSWDs with and without transient impairment of consciousness using routine EEGs of children with CAE. The focus of this study was to distinguish critical nodes of brain networks responsible for the cognitive impairment seen during absence seizures. By directly comparing ictal and subclinical events as well as the transition periods between pre-, during-, and post-ictal and subclinical periods, we have characterized relevant, complex neuronal networks and their dynamics.

Source reconstruction, as well as FC, revealed a complex network affecting virtually the entire brain. We have found that these two events have a similar pattern of sources and connections; however, the source power was significantly stronger during absences. Interestingly, we found significant source power differences not only during GSWDs but throughout transition periods i.e., pre-ictal to ictal to post-ictal as well.

These findings correlate with results from Guo et al. [5] which demonstrated that overall intensity of seizures correlates with behavioural impairment. We support the notion that GSWDs with impairment of consciousness are more electrophysiologically intense and show changes even during pre-ictal phases as well as throughout seizures.

### Source analysis

In this study, source reconstruction using the eLORETA technique revealed most importantly that, ictal and subclinical discharges have the same active sources however there is a marked difference in the intensity of the sources.

A comparison of time intervals for source analysis shows that during ictal and subclinical discharges, a more widespread source area is activated compared to pre- and post- time intervals (Supplementary Fig. 1). Since the during-interval consists of abnormal activity, the significant source power is higher compared to pre- and post-intervals and is seen to be localized in the pre-frontal, frontal cortex, and in deep subcortical regions for delta, theta, and alpha bands. However, beta band activity did not show deep sources. In our previous work [4], spectral analysis on the sensor level demonstrated similar results for the time effect for all frequency bands (1–30 Hz).

Comparisons of the two groups, ictal and subclinical, show similar sources, though ictal discharges have stronger source power in comparison. The significant sources were seen in brain regions such as the rectus gyrus, frontal inferior, medial, and superior gyruses, and neighbouring sources in the caudate and anterior cingulate regions (Supplementary Fig. 2). These findings can be interpreted to mean that ictal discharges have highly activated sources and therefore, there is a severe suspension of awareness, in contrast to subclinical discharges, where source activity is weaker and there is a maintenance of awareness. These significant brain regions have been previously seen to be involved in absence epilepsy [36].

In this study, we have also described the evolving complex network of sources seen in the transition periods of ictal and subclinical discharges. Most importantly for delta band as the transition from pre- to during- takes place for ictal/subclinical discharges, the source maximum was seen in caudate nuclei and putamen, as well as the thalamus, dorsolateral prefrontal cortex, parietal and temporal regions (Fig. 1A). Previous studies have demonstrated the involvement of the caudate-putamen in the modulation and propagation of GSWDs in absence epilepsy [37, 38]. A further transition from during- to post-ictal/subclinical depicts, the source dynamics evolving with significant differences in cortical brain regions such as the supplementary motor area (Fig. 1B). This suggests the probable role of suspension of certain motor activities during absence seizures. The transition dynamics demonstrate the involvement of a complex cortical-subcortical network of sources. Overall, we have observed the direct proportional relationship between the strength of source power and clinical symptoms.

Theta band (4–7 Hz) did not show any significant group or interaction effect in this study. In our previous sensor-level study [4] similar results were seen. We presume that in the slow wave activity of absence seizures, delta band, being more dominant, supresses the effects of theta band.

In a previous EEG based study, source analysis in 5 absence epilepsy patients revealed mesial frontal and orbital frontal cortex sources for spike and wave activity [39]. Using EEG data and standardized, low-resolution, brain electromagnetic tomography (sLORETA) [40] for CAE patients, another study depicted the maximal current source density of epileptiform discharges in the frontal lobe involving superior frontal gyrus, middle frontal gyrus, and medial frontal gyrus [36]. In this study, for alpha (8–12 Hz) (Fig. 1C, D) and beta bands (13–30 Hz) (Fig. 1E, F) the source maximum was seen in the frontal inferior orbital, frontal superior, medial and orbito-frontal regions. These results suggest the involvement of cortical frontal regions in generalized epilepsies and are consistent with previous studies [41, 42].

### Functional connectivity

FC at the source level was performed to explore neuronal networks during GSWDs with and without impairment of consciousness. It is well described in previous publications that the thalamus is one of the most significant brain regions involved in absences [17, 34, 35]. Therefore, we were particularly interested in exploring the relationship between the thalamus and the rest of the brain during ictal and subclinical EEG discharges.

We observed that the coherence between the thalamus and cortical regions, especially posterior brain regions, was significantly stronger during absences in all frequency bands (Supplementary Fig. 4). These differences were mainly prominent in the delta band (Supplementary Fig. 5). This was observed not only during absences but also before and after seizure intervals. Thus, we presume that the intensity of the connections between the thalamus and complex cortical structures described in this study is the driver of the ictal activity. Significant maximum coherence with the middle occipital region, as well as angular gyrus, precuneus, cingulum posterior, and supramarginal gyrus was observed for the delta band. The described regions are associated with brain functions such as language processing, consciousness, and visualization, further suggesting a correlation to the clinical symptoms observed in absence seizures. In a previous study, an investigation of the pre-treatment ictal connectivity in CAE patients in an EEG-fMRI and MEG study was performed and strong connections were found between the thalamus and posterior brain regions including the parietal, posterior cingulate, angular gyrus, precuneus and occipital regions for delta frequencies and frontal cortices for gamma frequencies [43]. Further, this study depicted decreased connectivity in the thalamus and basal ganglia and increased connectivity in the medial occipital cortex. Another study demonstrated activation of the thalamo-cortical network alongside suspension of the DMN [13].

It is observed that the connectivity between the thalamus and the cortical areas is much stronger during absence seizures and for transition periods (pre-, during- and post-) as compared to subclinical discharges. It can be presumed that the thalamus forms a strong network involving regions of the brain important for consciousness, and the DMN, leading to severe impairment of consciousness and clinical symptoms. However, for subclinical discharges, the intensity of connectivity in the described thalamo-cortical network does not reach the critical threshold for the suspension of consciousness. These network differences and critical brain regions identified for ictal and subclinical discharges further confirm our hypothesis and give insight into the consciousness system.

We can further speculate that connections between cortical regions themselves may be weaker, probably disrupting the large-scale information integration, or of a global neuronal workspace necessary for the emergence of consciousness. This however must further be investigated on a source level.

### Methodological considerations

Identifying the origin of brain activity based on surface EEG recordings has been one of the primary goals of solving the bioelectromagnetic inverse problem. The EEG inverse problem is an ill-posed problem since it has an unstable, non-unique solution. Various parametric and non-parametric methods have been developed to solve the bioelectromagnetic inverse problem [21]. In this study, the EEG inverse problem was solved using the eLORETA method [29]. This method was introduced as a generalized version of the previous LORETA and sLORETA algorithms [40]. This method is a non- parametric approach that, while based on the minimum norm inverse solution, still reduces the localization error of deep sources. Minimum norm estimates are used together with a regularization approach, which in the case of the eLORETA algorithm is the Tikhonov regularization [21, 22]. Using scalp EEG data eLORETA has shown promising results in reconstructing cortical as well as deep brain sources [44, 45]. This method has further been widely used for EEG source reconstruction in various other fields of research [46–48].

For FC analysis, the imaginary part of coherency was used to assess the networks on the source level. This was used to avoid the volume conduction problem, leading to false connectivity. When using EEG channels there is a tendency for multiple channels to pick up the activity of a particular source in the brain leading to the volume conduction problem. A method proposed by [49], used the imaginary part of coherency to interpret brain interactions. They described using the imaginary part of coherency as a robust method to overcome the volume conduction effect since it is not affected by it. The assumption used is that sources observed as a consequence of instantaneous activity are removed and the remaining imaginary part captures true brain interactions, which are only responsive to synchronizations of events that are time lagged to each other. Since volume conduction does not create any time lag; the imaginary part of coherency becomes unaffected by any kind of artifactual self-interaction. This method has been widely used as a measure of connectivity in various fields of research, to better understand brain interactions [50–52]

Several limitations of the present study should be considered. First, in this study the number of subjects as well as the number ictal and subclinical GSWDs analysed were small. To validate our findings, it would be important that the sample size be larger. Secondly, deep source localization can be difficult to mark the exact seizure onset zone, therefore, other methods could be used to validate whether the source localization was biased and how much this bias was. Lastly, it is important to note that other FC techniques such as directionality and graph analyses could be used to understand the network spread and other important parameters.

## Conclusion

To conclude, this study demonstrates significant differences between ictal and subclinical neuronal networks in CAE. Source activity revealed that ictal discharges have a stronger source intensity, as compared to subclinical discharges. As for FC, a complex, dynamical network was observed for ictal and subclinical discharges, involving brain regions associated with the consciousness system and components overlapping with the DMN. As seen for delta frequency band (1–3 Hz), the critical brain hubs in the network comprised of the posterior cingulate cortex (PCC), precuneus, angular gyrus, supramarginal gyrus, parietal superior, and occipital mid-regions. For ictal transition periods (pre- to during- to post-), the stronger connectivity between the thalamus and these brain regions, suggests the disruption of consciousness. While, for subclinical discharges, the intensity of connectivity being weaker in the described thalamo-cortical network, suggests that the network does not reach the critical threshold for the suspension of consciousness. The findings of this study are a step forward in expanding our understanding of the involved consciousness system in CAE, and the pathophysiology of these discharges. Furthermore, the methodological considerations of using eLORETA and FC based on the imaginary part of coherency for EEG data, suggests that these techniques are feasible to detect deep brain activity and may be considered in future diagnostic studies.

## Supplementary Information

Below is the link to the electronic supplementary material.

Supplementary file1 (DOCX 40 KB)

Supplementary file2 (PNG 2027 KB)

Supplementary file3 (PNG 867 KB)

Supplementary file4 (PNG 168 KB)

Supplementary file5 (PNG 1920 KB)

Supplementary file6 (PNG 254 KB)

## Data Availability

The EEG data that supports the findings of this study are available from the corresponding author upon request.
