# Supplementary material for: Neuronal networks underlying ictal and subclinical discharges in childhood absence epilepsy
Source: J Neurol. 2022 Nov 12;270(3):1402–15. doi: 10.1007/s00415-022-11462-8 (PMC9971098; doi:10.1007/s00415-022-11462-8)
Supplement: Supplementary file 1 — Supplementary file1 (DOCX 40 KB) [file 415_2022_11462_MOESM1_ESM.docx]

**Supplementary Information**

**Appendix 1**

**Source Localization**

***Time Effect***

For delta theta and alpha bands, the significant source maximum for during vs. pre and during vs. post intervals was observed in the rectus right (R), left (L) region (Supplementary Fig. 1). For theta and alpha bands neighbouring sources were seen in the frontal mid orbital R, frontal inferior triangular part R, and frontal inferior orbital R, regions. For beta band, the significant source maximum for during vs. pre and during vs. post interval was observed in the frontal orbital R, region.

***Group Effect***

For delta band, the source maximum for the significant difference between ictal vs. subclinical discharges was seen in the rectus R, with neighbouring sources being in the caudate L, frontal medial orbital R, and the cingulum anterior L region. For alpha band, the source maximum was seen in frontal inferior orbital L region, while for beta band it was seen in the frontal superior medial R, region (Supplementary Fig. 2).

***Interaction effect***

For delta band, the source maximum for significant differences were seen in the caudate and putamen for the transition interval pre-during, and in the supplementary motor area for the during-post period. The spread of significant source power was observed in thalamus, dorsolateral prefrontal cortex, parietal and temporal regions. For alpha band the source maximum was observed in frontal inferior orbital region for both transition intervals pre-during and during-post. In beta band, for pre-during and during-post transition intervals the source maximum was revealed in the frontal superior medial region.

**Appendix 2**

**2.1 Functional Connectivity- 30% thresholding**

Functional connectivity using the thalamus as the seed and the interaction effect, were used to determine the 30 % thresholding of brain regions. With thresholding the FC, the global network dynamics can be better observed.

**Delta Band-** For delta band, the transition intervals for ictal and subclinical discharges, pre- to during, comprises of the connectivity network consisting of regions from the the parietal lobe, occipital lobe, temporal lobe, limbic lobe and the cerebellum. Followed by the transition interval during- to post, involving brain regions from the parietal, occipital and limbic lobe. The detailed list of significan brain regions have been listed in Supplementary Tables 1 and 2.

**Alpha Band-** For alpha band, the transition interval pre- to during for ictal and subclinical discharges consists of the connectivity network involving brain regions from the frontal lobe, pareital lobe, temporal lobe and limbic lobe. Further for the transition period during- to post the network consisted of brain region from the frontal lobe, pareital lobe, temporal lobe, limbic lobe and insuala as well as the occipital lobe. Detailed brain regions have been listed in Supplementary Tables 3 and 4.

The tables demonstrate brain structures subject to statistically significant changes of FC with the thalamus, during the interaction effect transition periods for ictal and subclinical discharges.

**Table 1: delta band (1-3 Hz) FC with 30% thresholding- transition period pre-during**

| Brain structure | FC value | | | | Percentage of brain structure subject to statistically significant changes, % |
| --- | --- | --- | --- | --- | --- |
|  | Ictal discharges | | Subclinical discharges | |  |
|  | Pre- | During | Pre- | During |  |
| 1. Angular L | 0,2 | 0,34 | 0,2 | 0,26 | 98,5 |
| 1. Angular R | 0,2 | 0,32 | 0,2 | 0,25 | 98,4 |
| 1. Parietal Inf R | 0,21 | 0,33 | 0,2 | 0,26 | 96 |
| 1. Occipital Inf R | 0,19 | 0,32 | 0,2 | 0,24 | 95,7 |
| 1. Parietal Inf L | 0,21 | 0,32 | 0,2 | 0,26 | 91,9 |
| 1. Occipital Mid R | 0,2 | 0,34 | 0,21 | 0,26 | 88,5 |
| 1. Precuneus L | 0,2 | 0,31 | 0,19 | 0,25 | 85,2 |
| 1. Occipital Sup L | 0,19 | 0,31 | 0,19 | 0,26 | 85,1 |
| 1. Occipital Mid L | 0,19 | 0,32 | 0,2 | 0,25 | 80,8 |
| 1. SupraMarginal R | 0,19 | 0,29 | 0,19 | 0,25 | 80,2 |
| 1. Parietal Sup L | 0,2 | 0,3 | 0,19 | 0,25 | 79,5 |
| 1. SupraMarginal L | 0,19 | 0,29 | 0,19 | 0,25 | 77 |
| 1. Cerebellum Crus1 R | 0,19 | 0,34 | 0,21 | 0,25 | 67,7 |
| 1. Precuneus R | 0,2 | 0,32 | 0,19 | 0,26 | 67,2 |
| 1. Cingulum Post L | 0,19 | 0,31 | 0,17 | 0,24 | 65,7 |
| 1. Paracentral Lobule R | 0,2 | 0,31 | 0,19 | 0,25 | 64,8 |
| 1. Parietal Sup R | 0,2 | 0,31 | 0,19 | 0,26 | 64,3 |
| 1. Cingulum Post R | 0,19 | 0,33 | 0,19 | 0,25 | 52,2 |
| 1. Cuneus L | 0,2 | 0,35 | 0,2 | 0,27 | 49,2 |
| 1. Cerebellum Crus2 R | 0,19 | 0,34 | 0,21 | 0,24 | 49,1 |
| 1. Temporal Sup R | 0,19 | 0,33 | 0,2 | 0,25 | 45,7 |
| 1. Cerebellum Crus2 L | 0,19 | 0,34 | 0,21 | 0,25 | 40,9 |
| 1. Occipital Sup R | 0,19 | 0,3 | 0,19 | 0,25 | 40,3 |
| 1. Heschl L | 0,18 | 0,29 | 0,19 | 0,25 | 40 |
| 1. Temporal Mid R | 0,19 | 0,33 | 0,2 | 0,25 | 39,6 |
| 1. Cerebellum 6 R | 0,19 | 0,32 | 0,2 | 0,25 | 39,6 |
| 1. Cerebellum 6 L | 0,19 | 0,32 | 0,2 | 0,24 | 39,2 |
| 1. Cingulum Mid L | 0,2 | 0,31 | 0,19 | 0,24 | 38,8 |
| 1. Postcentral L | 0,2 | 0,31 | 0,2 | 0,25 | 38,3 |
| 1. Cerebellum 8 L | 0,19 | 0,33 | 0,21 | 0,25 | 38,3 |
| 1. Paracentral Lobule L | 0,2 | 0,32 | 0,2 | 0,26 | 36,1 |
| 1. Cerebellum Crus1 L | 0,19 | 0,34 | 0,21 | 0,25 | 32,9 |
| 1. Postcentral R | 0,2 | 0,3 | 0,2 | 0,25 | 32,1 |

*FC functional connectivity, R right, L left*

**Table 2: Delta band (1-3 Hz) FC with 30% thresholding- transition period during-post**

| Brain structure | FC value | | | | Percentage of brain structure subject to statistically significant changes, % |
| --- | --- | --- | --- | --- | --- |
|  | Ictal discharges | | Subclinical discharges | |  |
|  | During | Post- | During | Post |  |
| 1. Angular L | 0,33 | 0,18 | 0,25 | 0,21 | 89,3 |
| 1. Paracentral Lobule R | 0,31 | 0,18 | 0,25 | 0,21 | 78,3 |
| 1. SupraMarginal L | 0,31 | 0,17 | 0,24 | 0,21 | 62,1 |
| 1. Paracentral Lobule L | 0,31 | 0,18 | 0,25 | 0,21 | 57,6 |
| 1. Occipital Sup L | 0,33 | 0,18 | 0,25 | 0,21 | 50,8 |
| 1. Occipital Mid L | 0,34 | 0,18 | 0,25 | 0,21 | 50,2 |
| 1. Precuneus L | 0,32 | 0,18 | 0,25 | 0,21 | 44,5 |
| 1. Cingulum Mid L | 0,31 | 0,18 | 0,25 | 0,21 | 36 |
| 1. Cingulum Post L | 0,29 | 0,18 | 0,25 | 0,21 | 34,6 |
| 1. Parietal Inf L | 0,29 | 0,17 | 0,24 | 0,21 | 31,8 |

*FC functional connectivity, R right, L left*

**Table 3: Alpha Band (8-12 Hz) FC with 30% thresholding- transition period pre-during**

| Brain structure | FC value | | | | Percentage of brain structure subject to statistically significant changes, % |
| --- | --- | --- | --- | --- | --- |
|  | Ictal discharges | | Subclinical discharges | |  |
|  | Pre- | During | Pre- | During |  |
| 1. Supp Motor Area L | 0,15 | 0,32 | 0,18 | 0,3 | 94,6 |
| 1. Parietal Inf R | 0,14 | 0,28 | 0,19 | 0,28 | 92,5 |
| 1. Supp Motor Area R | 0,15 | 0,32 | 0,18 | 0,3 | 85,7 |
| 1. Heschl L | 0,15 | 0,3 | 0,18 | 0,29 | 59,6 |
| 1. Temporal Sup L | 0,16 | 0,31 | 0,18 | 0,29 | 54,4 |
| 1. Rolandic Oper L | 0,15 | 0,29 | 0,18 | 0,29 | 54 |
| 1. Cingulum Post L | 0,15 | 0,32 | 0,19 | 0,29 | 53,1 |
| 1. Cingulum Post R | 0,16 | 0,32 | 0,18 | 0,3 | 52,2 |
| 1. Frontal Sup L | 0,15 | 0,32 | 0,18 | 0,29 | 49,9 |
| 1. Cingulum Mid R | 0,15 | 0,32 | 0,19 | 0,29 | 46,2 |
| 1. Angular L | 0,14 | 0,29 | 0,19 | 0,28 | 42,9 |
| 1. Frontal Mid L | 0,15 | 0,32 | 0,19 | 0,29 | 41,7 |
| 1. Cingulum Mid L | 0,15 | 0,31 | 0,19 | 0,29 | 41,4 |
| 1. Parietal Inf L | 0,14 | 0,28 | 0,19 | 0,28 | 41,3 |
| 1. Parietal Sup R | 0,15 | 0,31 | 0,18 | 0,3 | 40,2 |
| 1. Frontal Sup Medial L | 0,15 | 0,31 | 0,19 | 0,29 | 37,8 |
| 1. SupraMarginal R | 0,15 | 0,3 | 0,18 | 0,29 | 36,9 |
| 1. Parietal Sup L | 0,14 | 0,29 | 0,19 | 0,28 | 35,5 |

*FC functional connectivity, R right, L left*

**Table 4: Alpha Band (8-12 Hz) FC with 30% thresholding- transition period during-post**

| Brain structure | FC value | | | | Percentage of brain structure subject to statistically significant changes, % |
| --- | --- | --- | --- | --- | --- |
|  | Ictal discharges | | Subclinical discharges | |  |
|  | During | Post- | During | Post- |  |
| 1. Frontal Sup Medial R | 0,33 | 0,15 | 0,3 | 0,18 | 98,2 |
| 1. Parietal Inf R | 0,36 | 0,15 | 0,32 | 0,18 | 97,8 |
| 1. Parietal Sup R | 0,35 | 0,14 | 0,32 | 0,18 | 97,6 |
| 1. Frontal Sup Medial L | 0,32 | 0,15 | 0,3 | 0,18 | 94,8 |
| 1. Supp Motor Area L | 0,35 | 0,15 | 0,31 | 0,18 | 93,6 |
| 1. Heschl R | 0,29 | 0,15 | 0,28 | 0,19 | 90,6 |
| 1. Supp Motor Area R | 0,35 | 0,15 | 0,31 | 0,18 | 84,7 |
| 1. Frontal Mid L | 0,33 | 0,15 | 0,3 | 0,18 | 82,5 |
| 1. Angular R | 0,34 | 0,15 | 0,32 | 0,18 | 81,3 |
| 1. Frontal Sup L | 0,33 | 0,15 | 0,3 | 0,18 | 81,1 |
| 1. Frontal Sup R | 0,34 | 0,15 | 0,3 | 0,18 | 78,2 |
| 1. Frontal Inf Tri R | 0,29 | 0,15 | 0,28 | 0,18 | 76,4 |
| 1. Cingulum Ant L | 0,3 | 0,15 | 0,28 | 0,18 | 75,9 |
| 1. Occipital Sup L | 0,31 | 0,15 | 0,3 | 0,18 | 75,4 |
| 1. Postcentral R | 0,36 | 0,15 | 0,31 | 0,18 | 72,9 |
| 1. Cingulum Ant R | 0,29 | 0,15 | 0,28 | 0,18 | 72,6 |
| 1. Occipital Sup R | 0,32 | 0,15 | 0,3 | 0,18 | 72,5 |
| 1. SupraMarginal R | 0,33 | 0,15 | 0,31 | 0,18 | 72 |
| 1. Rolandic Oper R | 0,29 | 0,15 | 0,28 | 0,18 | 70 |
| 1. Precuneus R | 0,35 | 0,15 | 0,32 | 0,18 | 70 |
| 1. Frontal Med Orb R | 0,31 | 0,15 | 0,29 | 0,18 | 61,4 |
| 1. Cingulum Post R | 0,32 | 0,16 | 0,3 | 0,18 | 57 |
| 1. Angular L | 0,32 | 0,15 | 0,31 | 0,19 | 56,8 |
| 1. Frontal Mid Orb R | 0,3 | 0,16 | 0,29 | 0,18 | 54,2 |
| 1. Occipital Mid L | 0,31 | 0,15 | 0,29 | 0,18 | 49,8 |
| 1. Temporal Pole Mid R | 0,31 | 0,15 | 0,3 | 0,19 | 49,5 |
| 1. Cingulum Post L | 0,33 | 0,16 | 0,3 | 0,18 | 49 |
| 1. Frontal Inf Orb R | 0,3 | 0,16 | 0,29 | 0,17 | 47,9 |
| 1. Precuneus L | 0,34 | 0,15 | 0,31 | 0,18 | 43,7 |
| 1. Precentral R | 0,36 | 0,15 | 0,31 | 0,18 | 43,2 |
| 1. Temporal Sup R | 0,31 | 0,15 | 0,29 | 0,18 | 38,5 |
| 1. Frontal Mid R | 0,31 | 0,15 | 0,29 | 0,18 | 36,8 |
| 1. Frontal Inf Oper R | 0,29 | 0,15 | 0,28 | 0,18 | 36,7 |
| 1. Parietal Sup L | 0,33 | 0,15 | 0,32 | 0,19 | 36,6 |
| 1. Cuneus L | 0,32 | 0,15 | 0,3 | 0,19 | 36,4 |
| 1. Cingulum Mid R | 0,32 | 0,15 | 0,3 | 0,18 | 36,3 |
| 1. Frontal Sup Orb R | 0,32 | 0,16 | 0,29 | 0,18 | 35,9 |
| 1. Insula R | 0,29 | 0,15 | 0,28 | 0,19 | 34,7 |
| 1. Cingulum Mid L | 0,33 | 0,15 | 0,3 | 0,18 | 33,5 |
| 1. Cuneus R | 0,31 | 0,15 | 0,29 | 0,19 | 32,3 |

*FC functional connectivity, R right, L left*

**Supplementary Figure Legends:**

**Supplementary Figure 1- Source analysis time effect**

For all frequency bands it was observed that the during-ictal/subclinical interval has stronger source power as compared to the pre- and post-ictal/subclinical intervals. The source power is wide spread, but stronger in frontal regions for all frequency bands. A) source power distribution for pre- to during-ictal/subclinical discharges for frequency bands delta, theta, alpha and beta B) source power distribution for the time interval during- to post- ictal/subclinical discharges for frequency bands delta, theta, alpha and bands.

**Supplementary Figure 2- Source analysis group effect**

For the group effect, ictal discharges have stronger source power as compared to subclinical discharges. The source power is widespread for all frequency bands. A) delta band, B) alpha band, C) beta band

**Supplementary Figure 3: Delta band statistical comparisons using t-test during ictal and subclinical discharges.**

T-test results showing statistically higher activities during ictal discharges compared to subclinical discharges.

**Supplementary Figure 4: Functional Connectivity time effect**

For all frequency bands it was observed that the during-ictal/subclinical interval has stronger imaginary part of coherency as compared to the pre- and post-ictal/subclinical intervals. With the thalamus as the seed, for delta and theta bands the functional connectivity can be seen prominant in frontal and posterior brain regions. As for alpha and beta bands the functional connectivity is widespread. A) distribution of imaginary part of coherency for the time intervals pre- to during-ictal/subclinical discharges for frequency bands delta, theta, alpha and bands. B) distribution of imaginary part of coherency for the time intervals during- to post-ictal/subclinical for frequency bands delta, theta, alpha and bands.
**Supplementary Figure 5: Functional connectivity group effect**

Significant group effect for delta band, depicting ictal discharges have stronger imaginary part of coherency as compared to subclinical discharges. With the thalamus as the seed, strong connections to angular gyrus, and supramarginal gyrus can be observed.
