# Supplementary figures and images for: Neuronal networks underlying ictal and subclinical discharges in childhood absence epilepsy
Source: J Neurol. 2022 Nov 12;270(3):1402–15. doi: 10.1007/s00415-022-11462-8 (PMC9971098; doi:10.1007/s00415-022-11462-8)

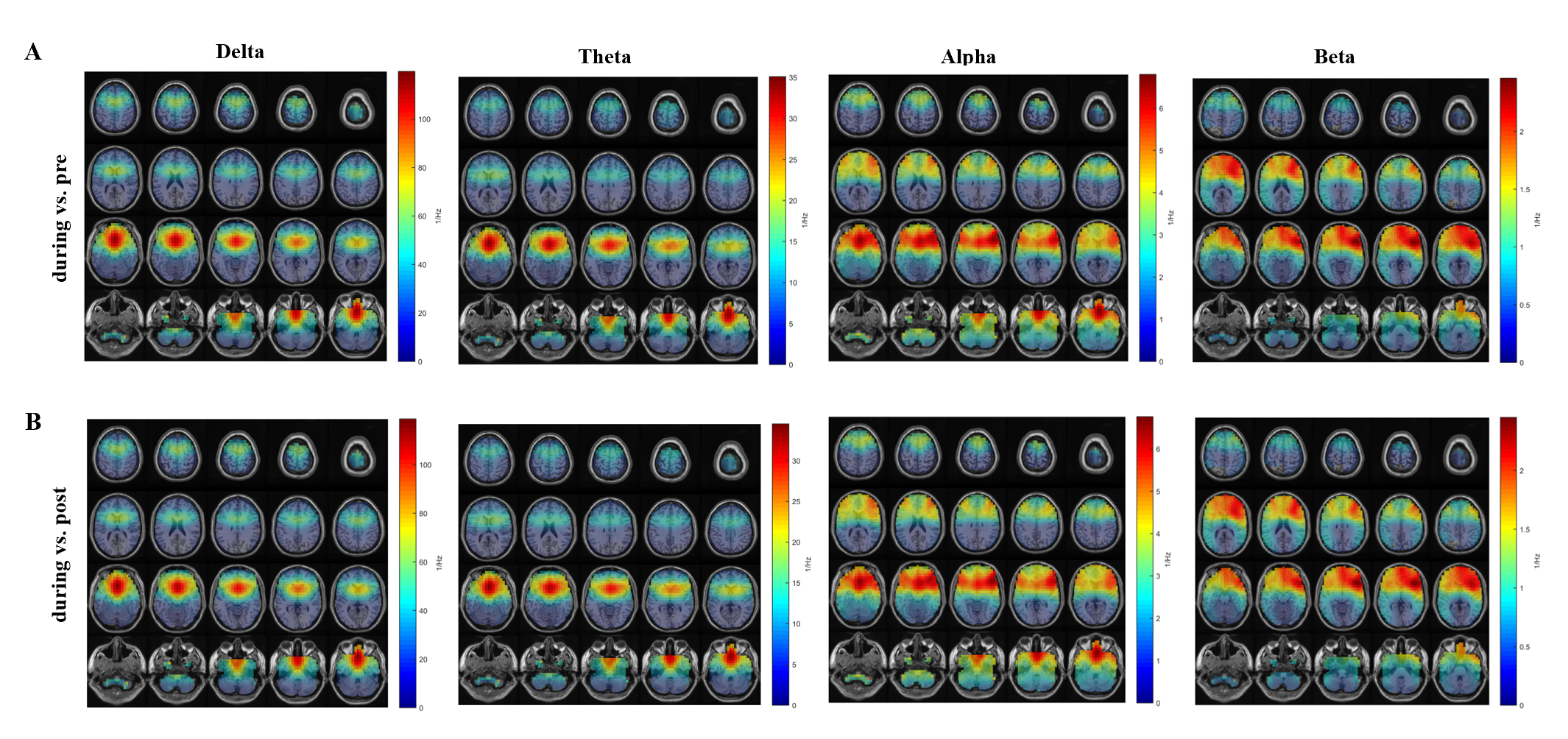

Supplement: Supplementary file 2 — Supplementary file2 (PNG 2027 KB) [file 415_2022_11462_MOESM2_ESM.png]

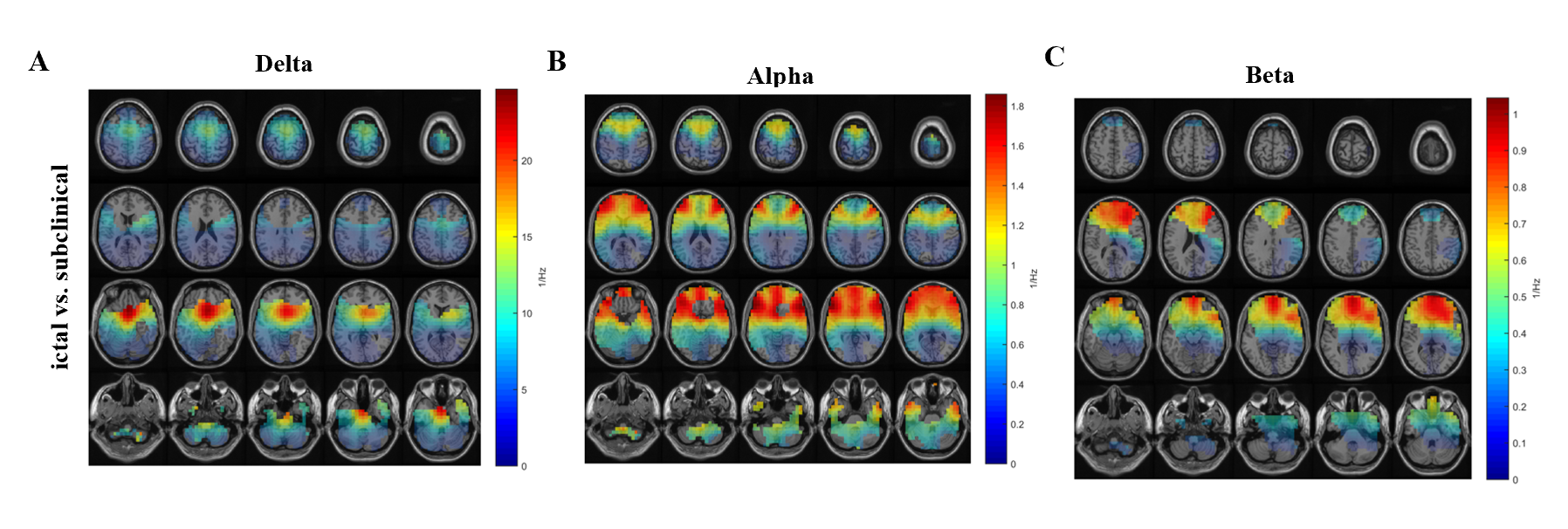

Supplement: Supplementary file 3 — Supplementary file3 (PNG 867 KB) [file 415_2022_11462_MOESM3_ESM.png]

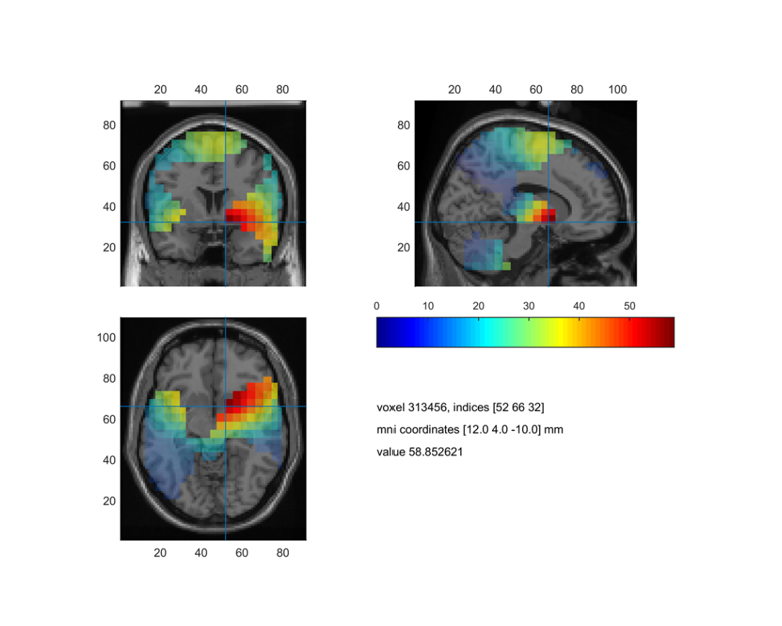

Supplement: Supplementary file 4 — Supplementary file4 (PNG 168 KB) [file 415_2022_11462_MOESM4_ESM.png]

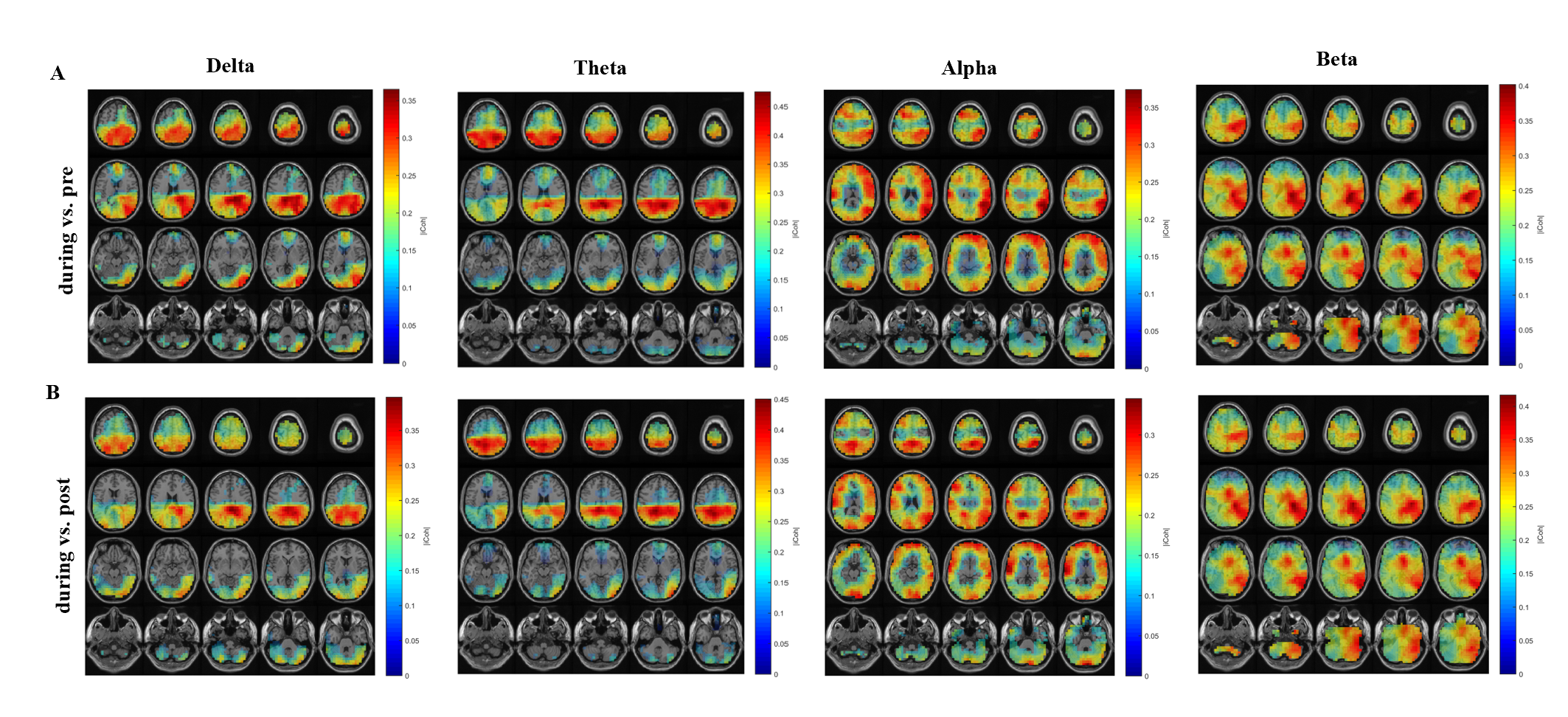

Supplement: Supplementary file 5 — Supplementary file5 (PNG 1920 KB) [file 415_2022_11462_MOESM5_ESM.png]

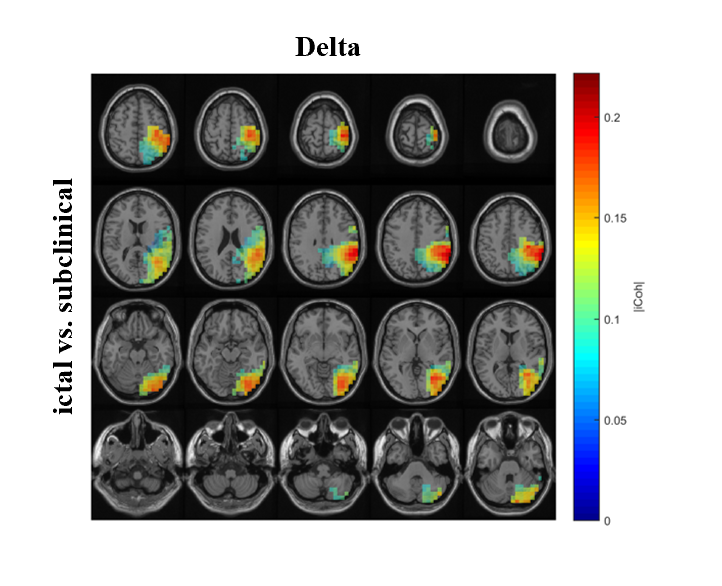

Supplement: Supplementary file 6 — Supplementary file6 (PNG 254 KB) [file 415_2022_11462_MOESM6_ESM.png]
